# Evaluation of inert gas rebreathing for determination of cardiac output: influence of age, gender and body size

**DOI:** 10.1038/s41440-018-0179-1

**Published:** 2018-12-18

**Authors:** Jessica E. Middlemiss, Alex Cocks, Kaido Paapstel, Kaisa M. Maki-Petaja, Sunita, Ian B. Wilkinson, Carmel M. McEniery, On behalf of the ACCT Study Investigators

**Affiliations:** 1 Division of Experimental Medicine and Immunotherapeutics, University of Cambridge, Cambridge, UK; 2 Department of Biochemistry, Centre of Excellence for Translational Medicine, University of Tartu, Tartu, Estonia

**Keywords:** Cardiac output, Inert gas rebreathing, Body size, Normative data

## Abstract

The aim of this study was to evaluate an inert gas rebreathing method (Innocor) for measurement of cardiac output and related haemodynamic variables and to provide robust normative data describing the influence of age, gender and body size on these variables. Four separate studies were conducted: measurement repeatability (study 1, *n* = 45); postural change (study 2, *n* = 40); response to submaximal cycling exercise (study 3, *n* = 20); and the influence of age, gender and body size (study 4, *n* = 1400). Repeated measurements of cardiac output, stroke volume and heart rate were similar, with low mean (±SD) differences (0.26 ± 0.53 L/min, 0 ± 11 mL and 2 ± 6beats/min, respectively). In addition, cardiac output and stroke volume both declined progressively from supine to seated and standing positions (*P* < 0.001 for both) and there was a stepwise increase in both parameters moving from rest to submaximal exercise (*P* < 0.001 for both). In study 4, there was a significant age-related decline in cardiac output and stroke volume in males and females, which remained significant after adjusting for body surface area (BSA, *P* < 0.001 for all comparisons). Both parameters were also significantly higher in those with high body mass index (BMI; *P* < 0.01 versus those with normal BMI for all comparisons), although indexing cardiac output and stroke volume to BSA reversed these trends. Inert gas rebreathing using the Innocor device provides repeatable measurements of cardiac output and related indices, which are sensitive to the effects of acute physiological manoeuvres. Moreover, inert gas rebreathing is a suitable technique for examining chronic influences such as age, gender and body size on key haemodynamic components of the arterial blood pressure.

## Introduction

Cardiac output (CO) is an important tool for monitoring the progression of, and response to therapy in, heart failure and related cardiac conditions. In addition, CO is a key determinant of the arterial blood pressure (BP). Together with peripheral vascular resistance (PVR), CO determines the mean level of BP within an individual, around which the systolic and diastolic pressures oscillate. Previous studies demonstrate that CO is elevated in individuals in the very early stages of hypertension [1–4] leading to the suggestion that an increased CO triggers a cascade of haemodynamic adaptations, ultimately resulting in sustained hypertension [5, 6]. CO is also strongly associated with body size [7, 8], which, in turn, is associated with BP [9, 10]. Therefore, although often overlooked in routine population screening, CO could be a valuable biomarker of future hypertension and cardiovascular risk, both in normal weight and overweight individuals.

Gold standard techniques for assessing CO, such as the direct Fick method and thermodilution, are highly invasive and unsuitable for routine screening in the general population. Such techniques are also likely to elicit a stress response, making it difficult to assess haemodynamic parameters in the ‘resting’ state. However, a number of non-invasive techniques for assessing CO are available, including cardiac magnetic resonance (CMR) imaging, transthoracic echocardiography (TTE) and inert gas rebreathing. Although considered the current gold standard, CMR is expensive, time consuming and lacks portability. Although TTE is widely used for clinical haemodynamic assessment, particularly in the field of heart failure, it is prone to technical errors relating to measurement of left ventricular outflow tract cross-sectional area and the velocity time integral, which may lead to significant under- or over-estimations of CO [11, 12]. Inert gas rebreathing, which is based on the Fick principle, allows the non-invasive determination of pulmonary blood flow and thus CO from the rate of blood soluble gas uptake by the lungs [13–15]. When coupled with a photoacoustic infrared spectrometer (Innocor device, Innovision A/S, Denmark), inert gas rebreathing provides comparable measures of pulmonary blood flow to those obtained with thermodilution [16–22], direct Fick [17–19, 21] and modified Fick [23, 24] methods, both at rest and during graded exercise. Moreover, the device provides reproducible measurements of CO at rest and during exercise in healthy individuals [22, 23, 25, 26] and in patients with cardiac indications [27, 28] or diffuse lung disease [23]. However, as yet, there are no normative data for measurements of CO and related indices using inert gas rebreathing in healthy individuals.

The major aim of the current study, therefore, was to provide age- and gender-specific normative data on CO and related indices, measured with inert gas rebreathing, in a large group of healthy individuals from across the adult age span. We also wished to examine the additional influence of body size on these measures. Finally, we wished to confirm the utility of inert gas rebreathing in our laboratory by assessing measurement repeatability and responses to postural change and submaximal exercise.

## Materials and methods

### Subjects

Four separate studies were conducted. Participants for all studies were drawn from our local volunteer databases (studies 1–3) and the Cambridge arm of the Anglo-Cardiff Collaborative Trial (ACCT; study 4), a large, community-based investigation of the determinants of BP and arterial stiffness across the adult age span [29]. All subjects in all studies were free of overt cardiovascular disease and medication. Local Research Ethics Committee approval was obtained and informed consent was given by all subjects.

### Study procedures

All studies were undertaken in the Vascular Research Clinic at Addenbrooke’s Hospital on a single visit, in a quiet, temperature-controlled environment. Subjects were asked to abstain from heavy exercise and alcohol for 24 h prior to testing. Subjects were also asked to refrain from eating large meals, ingesting caffeine or smoking for at least 4 h prior to arrival. Past medical and lifestyle history was checked and height and weight were recorded. Subjects were then familiarised with the test procedures, equipment and overall environment to ensure haemodynamic stability.

Haemodynamic measurements were undertaken after instruction of how to perform the rebreathing manoeuvre, using ambient air. All measurements were performed by the same investigator within each study, except for study 4, where measurements were made by four highly trained and experienced members of the ACCT study investigator team (LD, KM, JS, JW-S), following a uniform protocol.

Study 1—Repeatability: 45 healthy subjects (20 males, mean age 37 ± 10 years) were studied supine following at least 10 min of rest. Inert gas rebreathing was performed twice, with at least 5 min between readings to allow sufficient time for washout of the blood soluble gas.

Study 2—Effect of posture: 40 healthy subjects (21 males, mean age 27 ± 9 years) were studied supine, seated and standing, following at least 10 min of rest in each position. A further sub-study was conducted to examine whether the posture-induced changes in CO obtained with inert gas rebreathing were comparable with other methods. Therefore, in a further 10 healthy subjects (5 males, mean age 31 ± 9 years), measurements of CO and stroke volume (SV) were obtained with the Innocor device, as above, and with a non-invasive bioreactance method (Cheetah NICOM, Cheetah Medical, Delaware, USA). Further details of this method are included in the online data supplement. Subjects were studied supine and standing, following at least 10 min of rest. For both studies, the order in which each posture was studied was randomised between subjects.

Study 3—Effect of submaximal exercise: 20 healthy subjects (mean age 32 ± 11 years) were studied while resting, seated, on an upright calibrated cycle ergometer for at least 10 min prior to measurement. A single inert gas rebreathing manoeuver was performed at rest. Subjects then commenced bicycle exercise at 20 and 35 rpm, corresponding to approximately 12 and 25 watts, respectively, for 5 min at each workload. In the final minute of each workload, the inert gas rebreathing manoeuver was performed.

Study 4—Age- and gender-specific normative data: Data from a subset of healthy, treatment-naive subjects from the ACCT study population were analysed. Data from 50 males and 50 females with normal body mass index (BMI; <25 kg/m^2^) were selected at random from each decade of age, between the ages of 18 and 80 years. In order to examine any additional effect of body size on CO and related measures, data from a further 50 males and 50 females with higher BMI (>27 kg/m^2^) were selected at random from each decade of age. BMI was chosen as our index of body size because of its widespread use in clinical guidelines. All subjects were studied supine following at least 10 min of rest. Brachial BP was measured in the non-dominant arm, followed by a single inert gas rebreathing manoeuver.

### Haemodynamic measurements

#### Blood pressure

Brachial BP was assessed in duplicate (triplicate if >5 mm Hg difference), in the non-dominant arm, using a validated oscillometric sphygmomanometer (HEM-705CP; Omron Corporation, Japan).

#### CO and SV

Inert gas rebreathing was used to measure CO and SV. The technique is based on the principle that the rate of disappearance of a blood-soluble gas is proportional to the rate of pulmonary blood flow. A blood-insoluble gas is also measured to determine the lung volume from which the soluble gas disappears. The Innocor device utilises an oxygen-enriched gas mixture containing two foreign gases: nitrous oxide (blood soluble, 5%), and sulphur hexafluoride (blood insoluble, 1%). Study participants rebreathed the gas mixture from a rubber rebreathing bag over approximately 20–30 s, at a rate of 20 breaths/min. An infrared photoacoustic gas analyser embedded within the device measured gas concentrations at the mouthpiece. Individual values were then indexed to body surface area (BSA), as described previously [30]. PVR was then estimated using the formula: PVR (dynes/s/cm^5^) = mean arterial pressure (mm Hg) × 80/CO (L/min).

### Statistical analysis

Data were analysed using the SPSS software (version 23.0). Agreement between repeated readings in study 1 was assessed following the method of Bland and Altman [31]. Correlation between repeated readings was assessed with Pearson’s correlation coefficient (*r*). The effects of postural change and submaximal exercise were analysed using one-way analysis of variance (ANOVA) with repeated measures. The influence of age and body size on CO and related indices was assessed separately for males and females, using two-way ANOVA. The influence of gender was analysed separately for normal and high BMI groups using two-way ANOVA. Data are presented as means ± SD unless otherwise stated, and a *P* value of <0.05 was considered significant.

## Results

Subject characteristics for studies 1–3 are presented in Table 1.

**Table 1:** Descriptive characteristics for participants in studies 1, 2 and 3

Variable	Study 1	Study 2	Study 3
Age (years)	37 ± 10	27 ± 9	32 ± 11
Gender (M:F)	20:25	21:19	9:11
Height (m)	1.71 ± 0.08	1.73 ± 0.11	1.70 ± 0.09
Weight (kg)	72 ± 11	68 ± 14	71 ± 17
BMI (kg/m^2^)	24.7 ± 3.6	22.5 ± 3.3	24.4 ± 4.3

Data are means ± SD

*BMI* body mass index

### Study 1: Repeatability

**Fig. 1:**
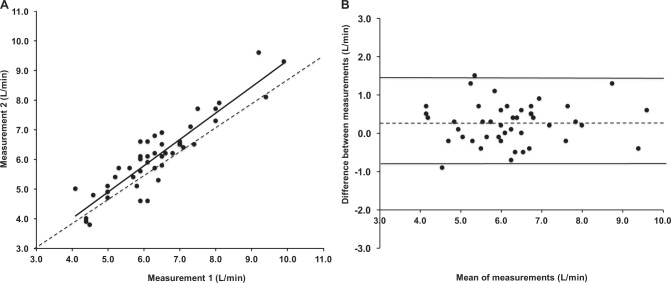
**a** Correlation between repeated readings of cardiac output. The dashed line indicates line of identity. **b** Bland–Altman plot showing agreement between repeated measurements of cardiac output

The means ± SD of the differences between repeated readings of CO, SV and heart rate were 0.26 ± 0.53 L/min, 0 ± 11 mL and 2 ± 6 beats/min, respectively (Supplementary Table 1). Repeated readings were highly correlated and Bland–Altman analyses did not show any evidence of systematic bias (Fig. 1a, b).

### Study 2: Postural changes

**Fig. 2:**
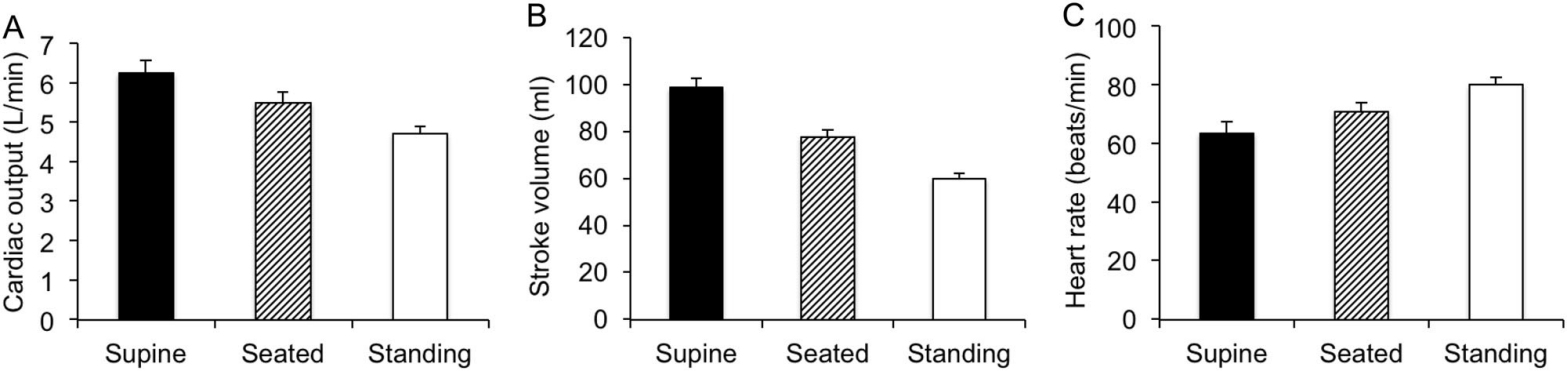
Cardiac output (**a**), stroke volume (**b**) and heart rate (**c**) in supine, seated and standing positions. Data are means ± SEM

Mean values of CO, SV and heart rate during each posture are shown in Supplementary Table 2, with data represented graphically in Fig. 2. CO and SV both declined progressively from supine to seated and standing positions, respectively (*P* < 0.001 for both), whereas heart rate increased from supine through to standing (*P* < 0.001).

#### Inert gas rebreathing versus bioreactance sub-study

Mean values of CO, SV and heart rate during supine and standing are shown for each method in Supplementary Table 3. Values were comparable between devices in each posture, as was the magnitude of change moving from supine to standing.

### Study 3: Submaximal exercise

**Fig. 3:**
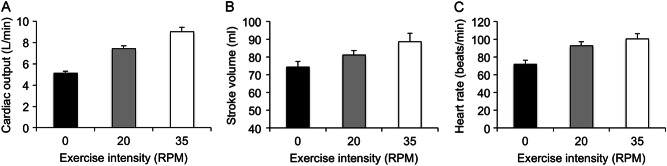
Cardiac output (**a**), stroke volume (**b**) and heart rate (**c**) at rest and during submaximal exercise. Data are means ± SEM

The mean values for CO, SV and heart rate during exercise are shown in Supplementary Table 4 with data represented graphically in Fig. 3. As expected, there was a stepwise increase in all three parameters moving from rest through the two exercise workloads (*P* < 0.001 for all).

### Study 4: Age- and gender-specific normative data and influence of body size

Data are presented by decade of age and level of BMI separately for males and females (Tables 2 and 3, respectively). In addition, Table 2 shows the overall influence of gender within each level of BMI. At all ages, CO and SV were significantly higher in males than in females, irrespective of level of BMI (*P* < 0.001 for all comparisons, Table 2). Indexing CO and SV to BSA attenuated these differences very slightly, but they remained significant.

**Table 2:** Haemodynamic variables per category of BMI, across the adult age span, in males

	BMI group	Age range (years)							Overall ANOVA		
		<20 (*n* = 100)	20–29 (*n* = 100)	30–39 (*n* = 100)	40–49 (*n* = 100)	50–59 (*n* = 100)	60–69 (*n* = 100)	70+ (*n* = 100)	Age *P*	BMI *P*	Gender^a^*P*
Age (years)	N	19 ± 1	23 ± 3	34 ± 3	44 ± 3	57 ± 2	64 ± 2	76 ± 5	<0.001	0.2	0.5
	H	18 ± 1	23 ± 3	34 ± 3	45 ± 3	56 ± 3	64 ± 3	74 ± 4			0.4
Height (m)	N	1.79 ± 0.06	1.79 ± 0.06	1.78 ± 0.07	1.79 ± 0.07	1.77 ± 0.06	1.75 ± 0.07	1.74 ± 0.06	<0.001	0.001	<0.001
	H	1.77 ± 0.06	1.76 ± 0.07	1.79 ± 0.07	1.77 ± 0.06	1.76 ± 0.07	1.74 ± 0.07	1.71 ± 0.09			<0.001
Weight (kg)	N	70.4 ± 6.0	69.2 ± 6.4	71.8 ± 7.8	73.8 ± 7.5	71.8 ± 7.0	71.0 ± 7.0	70.8 ± 6.0	0.001	<0.001	<0.001
	H	95.7 ± 11.7	93.1 ± 13.7	96.4 ± 11.2	92.7 ± 10.2	95.2 ± 13.3	88.9 ± 8.0	88.0 ± 12.8			<0.001
BMI (kg/m^2^)	N	22.0 ± 1.7	21.5 ± 1.7	22.7 ± 1.8	23.1 ± 1.4	22.8 ± 1.8	23.2 ± 1.3	23.3 ± 1.3	0.2	<0.001	<0.001
	H	30.6 ± 2.9	30.2 ± 4.2	30.0 ± 2.9	29.6 ± 2.5	30.7 ± 3.3	29.4 ± 2.0	29.9 ± 3.1			<0.001
BSA (m^2^)	N	1.88 ± 0.10	1.87 ± 0.10	1.89 ± 0.13	1.92 ± 0.13	1.89 ± 0.11	1.86 ± 0.12	1.85 ± 0.11	<0.001	<0.001	<0.001
	H	2.12 ± 0.14	2.09 ± 0.16	2.15 ± 0.15	2.10 ± 0.13	2.11 ± 0.17	2.04 ± 0.12	2.00 ± 0.19			<0.001
Systolic BP (mm Hg)	N	123 ± 12	124 ± 14	127 ± 15	131 ± 15	127 ± 16	133 ± 16	140 ± 17	<0.001	<0.001	<0.001
	H	133 ± 12	134 ± 15	140 ± 18	133 ± 15	133 ± 14	141 ± 14	144 ± 17			<0.001
Diastolic BP (mm Hg)	N	73 ± 9	75 ± 9	78 ± 10	84 ± 10	78 ± 11	81 ± 10	80 ± 10	<0.001	<0.001	<0.001
	H	81 ± 9	84 ± 11	87 ± 11	86 ± 10	86 ± 9	87 ± 8	82 ± 9			<0.001
Heart rate (beats/min)	N	67 ± 10	63 ± 10	60 ± 12	65 ± 11	64 ± 10	66 ± 11	64 ± 10	0.002	0.01	0.004
	H	71 ± 12	68 ± 11	65 ± 13	65 ± 9	65 ± 11	65 ± 11	65 ± 8			0.002
Cardiac output (L/min)	N	8.1 ± 1.3	7.1 ± 1.3	6.8 ± 1.5	6.6 ± 1.7	5.8 ± 1.5	5.1 ± 1.9	4.3 ± 0.9	<0.001	<0.001	<0.001
	H	8.7 ± 1.8	8.3 ± 1.9	7.6 ± 1.7	6.8 ± 1.6	5.7 ± 1.2	5.3 ± 0.9	4.3 ± 0.9			<0.001
Cardiac index (L/min/m^2^)	N	4.3 ± 0.7	3.8 ± 0.6	3.6 ± 0.8	3.5 ± 0.8	3.1 ± 0.8	2.7 ± 1.0	2.3 ± 0.5	<0.001	0.02	0.007
	H	4.1 ± 0.9	4.0 ± 0.9	3.5 ± 0.7	3.2 ± 0.8	2.7 ± 0.6	2.6 ± 0.5	2.2 ± 0.5			0.003
Stroke volume (mL)	N	101 ± 23	98 ± 24	104 ± 27	98 ± 26	89 ± 26	78 ± 30	67 ± 17	<0.001	0.005	<0.001
	H	114 ± 31	113 ± 31	112 ± 27	98 ± 21	88 ± 22	81 ± 18	67 ± 14			<0.001
Stroke volume index (mL/m^2^)	N	54 ± 11	52 ± 11	55 ± 14	51 ± 13	47 ± 13	42 ± 15	36 ± 10	<0.001	0.01	<0.001
	H	54 ± 16	54 ± 15	52 ± 13	47 ± 10	42 ± 10	40 ± 9	34 ± 8			<0.001
Peripheral vascular resistance (dynes/s/cm^5^)	N	847 ± 187	986 ± 205	1097 ± 347	1196 ± 345	1341 ± 390	1744 ± 712	1872 ± 435	<0.001	0.4	<0.001
	H	839 ± 203	911 ± 197	1058 ± 235	1208 ± 298	1427 ± 336	1601 ± 422	1888 ± 570			<0.001

Data are means ± SD

*BMI* body mass index, *BSA* body surface area, *BP* blood pressure, *N* normal BMI (<25 kg/m^2^), *H* higher BMI (>27 kg/m^2^)

^a^Values in the final column represent the influence of gender within each level of BMI

**Table 3:** Haemodynamic variables per BMI category, across the age span, in females

	BMI group	Age group (years)							Age *P*	BMI *P*
		<20 (*n* = 100)	20–29 (*n* = 100)	30–39 (*n* = 100)	40–49 (*n* = 100)	50–59 (*n* = 100)	60–69 (*n* = 100)	70+ (*n* = 100)		
Age (years)	N	19 ± 1	22 ± 2	34 ± 3	45 ± 3	56 ± 3	64 ± 3	75 ± 4	<0.001	0.9
	H	18 ± 1	24 ± 3	35 ± 3	45 ± 3	55 ± 3	64 ± 3	74 ± 4		
Height (m)	N	1.65 ± 0.07	1.65 ± 0.06	1.66 ± 0.07	1.65 ± 0.07	1.62 ± 0.06	1.61 ± 0.07	1.60 ± 0.07	<0.001	0.02
	H	1.66 ± 0.07	1.64 ± 0.06	1.63 ± 0.08	1.62 ± 0.06	1.62 ± 0.06	1.59 ± 0.10	1.60 ± 0.07		
Weight (kg)	N	57.8 ± 7.1	59.9 ± 6.4	60.0 ± 8.5	61.5 ± 6.1	58.5 ± 6.8	57.4 ± 7.9	57.3 ± 6.7	0.01	<0.001
	H	85.5 ± 11.9	82.8 ± 13.7	83.6 ± 13.4	83.2 ± 13.1	81.3 ± 10.5	80.3 ± 10.7	78.95 ± 13.0		
BMI (kg/m^2^)	N	21.1 ± 2.0	21.9 ± 1.7	21.6 ± 2.2	22.5 ± 1.5	22.2 ± 2.1	22.2 ± 2.2	22.3 ± 1.8	0.7	<0.001
	H	31.0 ± 3.0	30.6 ± 4.3	31.3 ± 4.0	31.8 ± 5.0	31.0 ± 3.9	31.9 ± 4.8	30.7 ± 3.7		
BSA (m^2^)	N	1.63 ± 0.12	1.66 ± 0.11	1.67 ± 0.14	1.68 ± 0.11	1.62 ± 0.11	1.59 ± 0.14	1.59 ± 0.12	<0.001	<0.001
	H	1.93 ± 0.16	1.89 ± 0.16	1.89 ± 0.17	1.89 ± 0.14	1.86 ± 0.13	1.82 ± 0.15	1.82 ± 0.17		
Systolic BP (mm Hg)	N	109 ± 10	112 ± 13	114 ± 15	113 ± 13	125 ± 18	131 ± 15	139 ± 20	<0.001	<0.001
	H	119 ± 17	123 ± 13	127 ± 17	127 ± 16	130 ± 16	138 ± 16	141 ± 20		
Diastolic BP (mm Hg)	N	71 ± 9	73 ± 10	75 ± 9	72 ± 10	78 ± 10	79 ± 9	78 ± 10	<0.001	<0.001
	H	77 ± 8	82 ± 12	83 ± 14	83 ± 10	82 ± 9	83 ± 11	80 ± 8		
Heart rate (beats/min)	N	71 ± 15	67 ± 12	65 ± 10	65 ± 10	66 ± 9	66 ± 9	66 ± 10	0.006	0.005
	H	72 ± 10	70 ± 11	68 ± 11	70 ± 10	68 ± 10	67 ± 10	66 ± 10		
Cardiac output (L/min)	N	6.5 ± 1.4	6.2 ± 1.4	5.9 ± 1.4	5.5 ± 1.0	4.6 ± 1.3	4.0 ± 0.9	3.6 ± 0.8	<0.001	<0.001
	H	7.6 ± 1.7	6.9 ± 1.7	6.2 ± 1.3	6.1 ± 1.2	4.8 ± 0.9	4.5 ± 1.1	4.1 ± 1.1		
Cardiac index (L/min/m^2^)	N	4.0 ± 0.9	3.8 ± 0.9	3.5 ± 0.7	3.3 ± 0.6	2.8 ± 0.7	2.5 ± 0.5	2.3 ± 0.5	<0.001	0.02
	H	3.9 ± 0.8	3.6 ± 0.8	3.3 ± 0.6	3.2 ± 0.6	2.6 ± 0.5	2.5 ± 0.6	2.3 ± 0.6		
Stroke volume (mL)	N	79 ± 18	84 ± 22	86 ± 25	81 ± 18	65 ± 15	59 ± 14	55 ± 12	<0.001	<0.001
	H	89 ± 21	89 ± 17	86 ± 19	81 ± 16	69 ± 13	66 ± 18	64 ± 18		
Stroke volume index (mL/m^2^)	N	48 ± 10	51 ± 13	51 ± 14	49 ± 10	40 ± 9	37 ± 8	35 ± 8	<0.001	<0.001
	H	46 ± 10	47 ± 9	45 ± 10	43 ± 8	37 ± 7	36 ± 10	35 ± 10		
Peripheral vascular resistance (dynes/s/cm^5^)	N	1028 ± 251	1106 ± 346	1198 ± 341	1275 ± 322	1747 ± 653	1985 ± 626	2260 ± 740	<0.001	0.2
	H	952 ± 273	1093 ± 347	1272 ± 325	1276 ± 322	1670 ± 502	1930 ± 739	2053 ± 631		

Data are means ± SD. Data on the influence of gender appear in the final column of Table 2

*BMI* body mass index, *BSA* body surface area, *BP*  blood pressure, *N* normal BMI (<25 kg/m^2^), *H* higher BMI (>27 kg/m^2^)

There was a significant decline in CO across the age span in both males and females (*P* < 0.001 for both). There was also a significant decline in SV across the age span in males and females (*P* < 0.001 for both) and heart rate (*P* = 0.002, males; and *P* = 0.006, females), although the magnitude of the difference in heart rate between the youngest and oldest age groups was small and varied overall across the age span. In contrast, there was a significant increase in systolic and diastolic BPs and PVR across the age span in both males and females (*P* < 0.001 for all comparisons), although diastolic BP tended to decline in older individuals. The age-related trends in CO and SV remained significant after adjusting for BSA in both males and females (*P* < 0.001 for all comparisons).

**Fig. 4:**
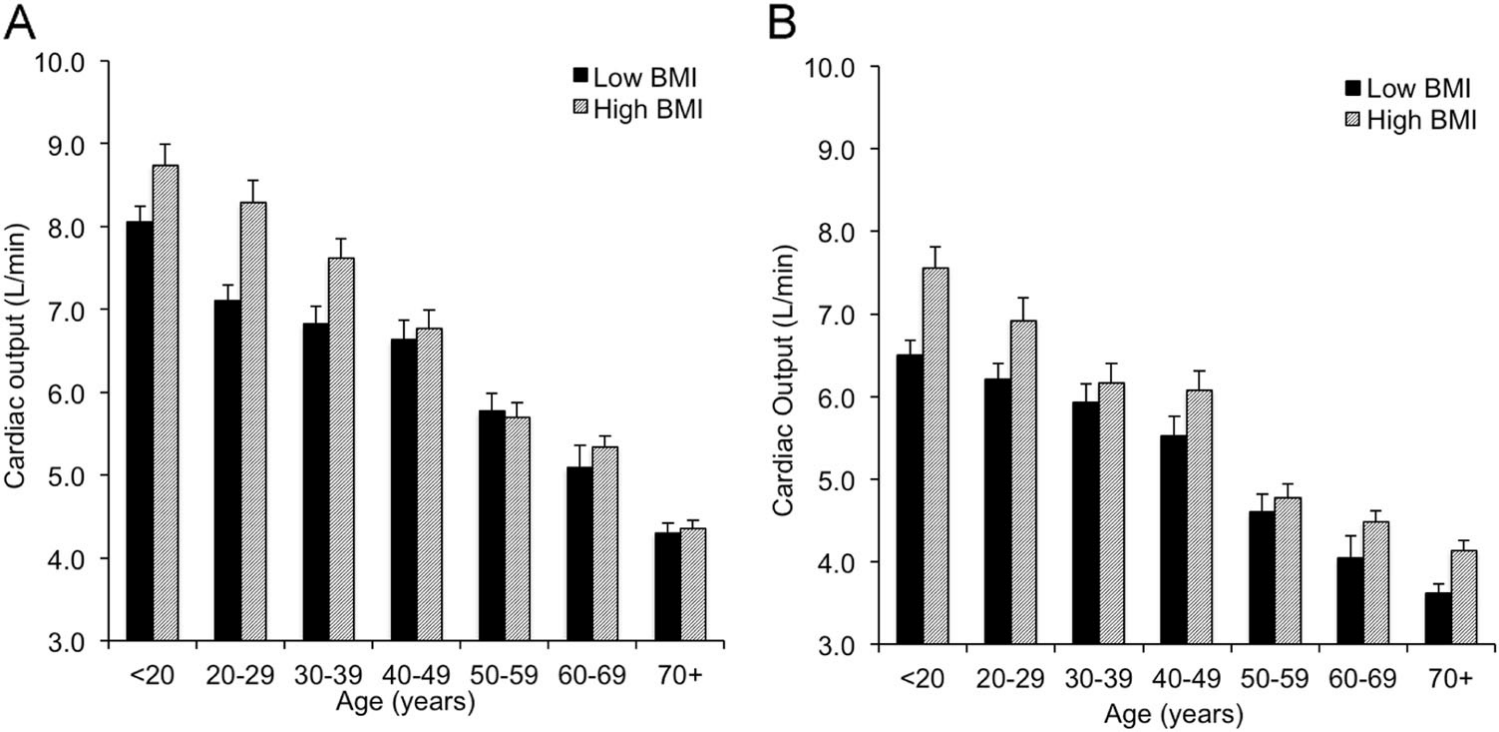
Influence of age and body size on cardiac output in males (**a**) and females (**b**). Data are means ± SEM

**Fig. 5:**
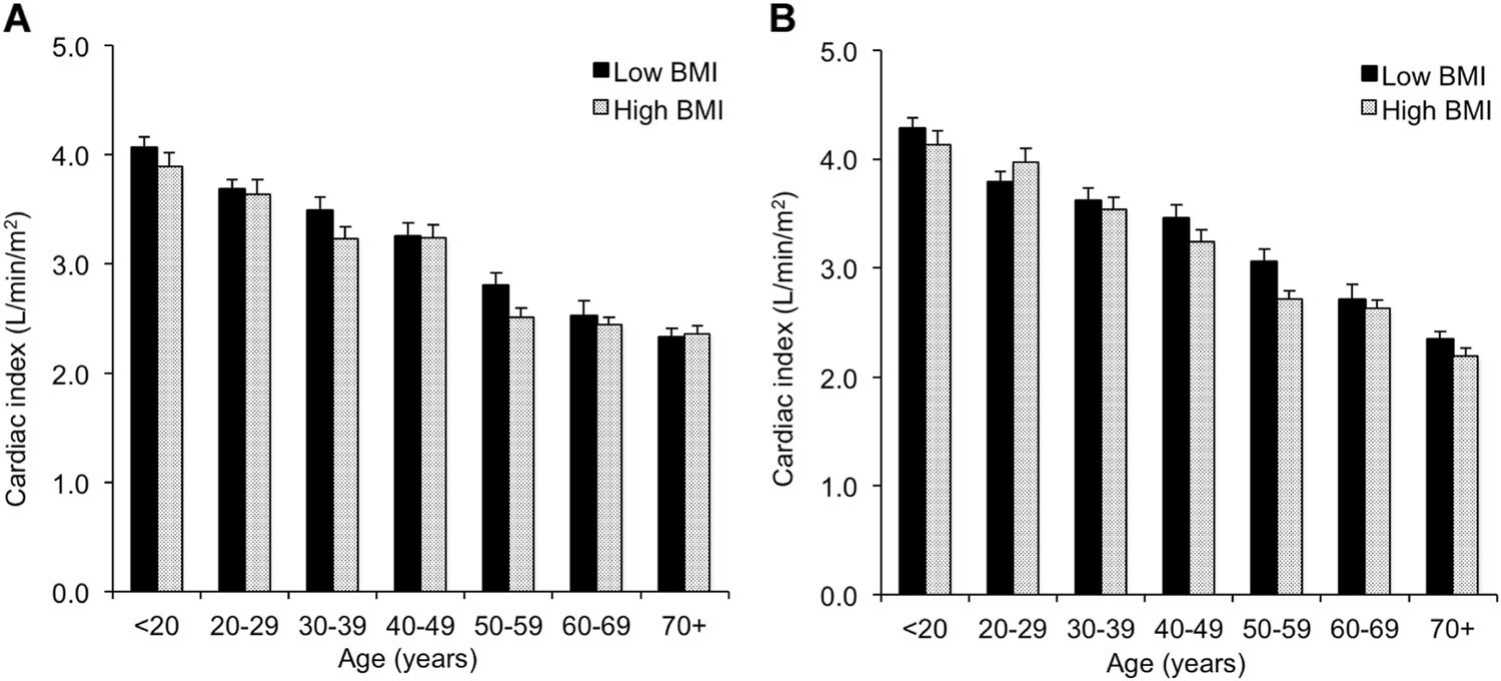
Influence of age and body size on cardiac index in males (**a**) and females (**b**). Data are means ± SEM

Comparing subjects with normal versus high BMI revealed a significantly higher level of CO overall in males and females with high BMI than those with normal BMI (*P* < 0.001 for both). This pattern appeared to be more marked in younger individuals (Figs. 4 and 5), although the interaction term (age group×BMI group) was significant only in males (*P* = 0.02, data not shown). Similarly, SV was higher overall in males and females with high BMI (*P* = 0.005 and *P* < 0.001, respectively), as were systolic and diastolic BPs (*P* < 0.001 for all comparisons) with no significant interaction with age group, indicating that these trends remained apparent across the age span. Interestingly, indexing the CO and SV to BSA reversed these trends, such that cardiac index and SV index were significantly higher in males and females with normal BMI versus those with high BMI (cardiac index: *P* = 0.02 and *P* = 0.015; SV index: *P* = 0.013 and *P* < 0.001, for males and females, respectively). There were no significant interactions with age group, indicating that these trends remained apparent across the age span.

## Discussion

We have demonstrated that inert gas rebreathing using the Innocor device provides repeatable measurements of CO and related indices, which are sensitive to the effects of physiological manoeuvres such as postural change and submaximal exercise. In addition, we have provided robust, age- and gender-specific normative data on CO in healthy adults. In doing so, we have demonstrated that CO and SV decline significantly across the adult age span in males and females and that body size exerts a significant impact on these haemodynamic measures.

Although we did not assess the performance of the Innocor against a ‘gold-standard’ invasive or non-invasive method in the current study, such data have been reported previously. The Innocor provides comparable measures of pulmonary blood flow to those obtained with thermodilution [16–22], direct Fick [17–19, 21] and modified Fick [23, 24] methods, both at rest and during graded exercise. In addition, the Innocor has been compared with the non-invasive gold-standard CMR in patients with indications for CMR imaging [28] and in pulmonary patients and matched controls [32], with both studies demonstrating good agreement, even in the presence of obstructive or restrictive pulmonary disease. Furthermore, the device has been used successfully in patients with heart failure, both at rest and during graded exercise [33], with reproducible results and good agreement with both invasive (Fick, thermodilution) [17, 19, 34] and non-invasive (CMR) [34] gold standards.

Our data on the repeatability of CO estimations are in agreement with previous studies of measurement repeatability performed in healthy subjects with the Innocor device, in terms of mean values (5.27 to 7.25 L/min), bias (0.00 to 0.32 L/min) and confidence intervals (−0.88 to 0.88 L/min) [22, 23, 25, 26]. In addition, we have demonstrated that the device is sensitive to acute physiological perturbation. Highest values were observed with subjects resting supine and lowest values with subjects standing. These trends were not unexpected, since venous return (preload) is relatively higher in supine versus standing positions due to the greater influence of gravity during standing. Moreover, we have demonstrated that the changes in CO and SV observed with the Innocor device were similar in magnitude to those observed with a different device (Cheetah NICOM), which is based on a bioreactance technique. Therefore, the data demonstrate that (1) the Innocor device is sufficiently sensitive to detect the effects of altered venous return through a manoeuvre such as postural change and (2) that posture is an important consideration when undertaking measurements of CO and interpreting values from other studies. Submaximal exercise also produced the expected increases in CO, SV and heart rate. Previous studies have examined inert gas rebreathing with the Innocor during maximal exercise in healthy subjects [25, 26, 35] and in patients with heart failure [33]. However, the intensity of our exercise protocol was relatively light, equating to brisk walking in healthy individuals, since we were interested in assessing the sensitivity of the system across exercise intensities that might be incurred by the activities of daily living, rather than under dedicated exercise testing conditions per se.

The utility of inert gas rebreathing has been evaluated in a number of different patient populations, but, as yet, normative data on CO obtained with this technique have not previously been available in a sufficiently large number of healthy adults. Our data demonstrate that CO and SV are higher in males than in females at all ages and that CO declines significantly over the adult age span in both sexes. In contrast, PVR increased significantly in both sexes. The age-related decline in CO in males and females appeared to be due, predominantly, to a decline in SV, since the overall difference in heart rate between younger and older age groups was small (2–4 beats/min), while differences between individual age groups were also variable. Previous studies have examined differences in CO across the age span, although with mixed results, possibly due to variations in measurement techniques and the inclusion of relatively small numbers of subjects. Nevertheless, studies using the direct Fick [36–38], dye dilution [39–41] and radiocardiography [42] methods have demonstrated significantly lower values of CO, SV and their indexed values in older compared with younger subjects, although other studies using similar techniques have not confirmed these trends [43, 44]. Non-invasive methods, more suitable for use in the general population, have also yielded conflicting results, with significant age-related differences in CO reported with two-dimensional (2D) echocardiography [45] and cardiac magnetic resonance imaging [46, 47] but not with transthoracic electrical bioimpedance [48].

Interestingly, the age-related trends in CO and SV observed in the current study were not related to a smaller body size in older individuals, since indexing to BSA still revealed a marked age-related decline in each variable. Possible mechanisms underlying our observations include a decline in left ventricular volume across the age span, which is lower in females, even after adjusting for differences in body size [49], inversely associated with age [45–47, 49, 50] and has previously been related to age-related declines in CO and SV, assessed with 2D echocardiography [45]. Alternatively, the CO and SV are strongly linked with the metabolic demand for oxygen [51, 52], which declines significantly with age, independently of changes in body size or composition [53, 54]. Unfortunately, neither left ventricular volume nor metabolic activity was assessed in the current study, making it impossible to conclude whether or the extent to which either mechanism might explain our observations. In contrast, we observed a significant increase in BP across the age span. This was not unexpected and has been demonstrated previously [55], but our data support previous observations [29, 56] that haemodynamic mechanisms other than CO are likely to underlie the significant increase in BP seen with age.

We also noted that both CO and SV were significantly higher in individuals with higher BMI versus those with normal BMI in both males and females. Systolic and diastolic BPs were also significantly higher in individuals with higher BMI. These trends appeared to be most marked in younger individuals (i.e., <40 years) in keeping with the notion that CO and SV appear to be the key haemodynamic determinants of elevated BP in younger individuals [3, 4], albeit with different underlying mechanisms [8]. Indexing these variables to BSA actually reversed these differences such that cardiac index and SV index were significantly higher in males and females with normal BMI versus those with high BMI. These observations suggest either that our adjustment for BSA may not have normalised the data optimally or that lean individuals may have an inherently higher CO when differences in body size are taken into account. CO and SV are typically indexed to BSA since this variable is thought to be proportional to metabolic rate, consistent with the idea that BSA regulates body temperature [57]. However, this method of normalisation may be misleading because the BSA is heavily influenced by the presence of adipose tissue, which has a lower metabolic demand and increases more than lean tissue mass in obesity [7, 58]. Indeed, CO and SV are more strongly associated with lean tissue mass than other indices of body size [52, 59], indicating that indexing to lean tissue mass may be more appropriate.

A potential limitation of the inert gas rebreathing method is the need for participants to actively engage with the rebreathing manoeuvre, which might present difficulties in very elderly or frail individuals. Thus clear and precise operator instructions are necessary. Moreover, the presence of pulmonary disease leading to uneven ventilation or the presence of any significant intrapulmonary shunt may result in erroneous determinations of pulmonary blood flow, and thus CO. In addition, our data are based on cross-sectional observations and longitudinal data would better inform the extent to which ageing influences haemodynamic variables. It was also beyond the scope of the present study to examine detailed measurements of ventricular volume, metabolic rate or body composition, but inclusion of such data may have provided valuable mechanistic insights concerning the trends observed in this study. Nevertheless, we have demonstrated that inert gas rebreathing with the Innocor device provides repeatable measurements of CO, which are sensitive to acute physiological perturbations and chronic influences of age, gender and body size. Further studies examining the contribution of CO and other haemodynamic variables to age-related changes in BP are warranted.

## Electronic supplementary material

Supplementary Information
