# Supplementary material for: Evaluation of inert gas rebreathing for determination of cardiac output: influence of age, gender and body size
Source: Hypertens Res. 2018 Dec 18;42(6):834–44. doi: 10.1038/s41440-018-0179-1 (PMC8076049; doi:10.1038/s41440-018-0179-1)
Supplement: Supplementary file 1 — Supplementary Information [file 41440_2018_179_MOESM1_ESM.doc]

**Supplementary Information**

**Evaluation of inert gas rebreathing for determination of cardiac output:**

**Influence of age, gender and adiposity**

Jessica E Middlemiss*^1^

Alex Cocks*^1^

Kaido Paapstel^2^

Kaisa Maki-Petaja^1^

Sunita^1^

Ian B Wilkinson^1^

Carmel M McEniery^1^

*On behalf of the ACCT Study Investigators*

*These authors contributed equally to this work

^1^Division of Experimental Medicine and Immunotherapeutics

University of Cambridge

Cambridge, UK

^2^Department of Biochemistry, Centre of Excellence for Translational Medicine

University of Tartu

Tartu, Estonia

**Short Title**: Inert gas rebreathing and cardiac output

**Correspondence:**

Dr Carmel McEniery

Division of Experimental Medicine and Immunotherapeutics

University of Cambridge

Addenbrooke’s Hospital, Box 98

Cambridge, UK

Tel: +44 (0)1223 217564

Fax: +44 (0)1223 216893

Email: cmm41@cam.ac.uk

**Bioreactance Method**

In a sub-study of Study 2, cardiac ouput was assessed with a non-invasive bioreactance method (Cheetah NICOM, Cheetah Medical, Delaware, USA). This method uses an electrical signal processing approach to analyze the frequency of relative phase shifts of electrical current applied across the thorax, using dual sensor pads placed on the upper body and abdomen. Data were sampled continuously and averaged over 30 second intervals.

**Supplementary Table 1. Repeatability of measurements of cardiac output, stroke volume and heart rate with inert gas rebreathing.**

|  | **Reading 1** | **Reading 2** | **Difference** | **CI** | **r** | **P** |
| --- | --- | --- | --- | --- | --- | --- |
| Cardiac output (L/min) | 6.36±1.29 | 6.10±1.26 | 0.26±0.53 | 0.10-0.42 | 0.91 | <0.001 |
| Stroke volume (ml) | 99±22 | 99±21 | 0±11 | -3-3 | 0.87 | <0.001 |
| Heart rate (beats/min) | 65±10 | 63±10 | 2±6 | 1-4 | 0.85 | <0.001 |

Data are means±SD. CI = Confidence intervals.

**Supplementary Table 2. Effect of posture on measurements of cardiac output, stroke volume and heart rate obtained with inert gas rebreathing.**

| **Parameter** | **Supine** | **Seated** | **Standing** | **Overall P** |
| --- | --- | --- | --- | --- |
| Cardiac output (L/min) | 6.3±2.1 | 5.5±1.9 | 4.7±1.1 | P<0.001 |
| Stroke volume (ml) | 99±25 | 78±20 | 60±14 | P<0.001 |
| Heart rate (beats/min) | 63±14 | 71±13 | 80±15 | P<0.001 |

Data are means±SD.

**Supplementary Table 3. Effect of posture on measurements of cardiac output, stroke volume and heart rate obtained with inert gas rebreathing (Innocor) and bioreactance (Cheetah) methods.**

|  | **Innocor** | **Cheetah** | **Difference** | **P** |
| --- | --- | --- | --- | --- |
| **Supine measures** |  |  |  |  |
| Cardiac output (L/min) | 7.26±1.50 | 7.29±0.73 | 0.03±1.37 | 0.9 |
| Stroke volume (ml) | 104±27 | 110±22 | 6±16 | 0.3 |
| Heart rate (beats/min) | 71±12 | 70±13 | -2±5 | 0.4 |
| **Standing measures** |  |  |  |  |
| Cardiac output (L/min) | 4.90±0.94 | 4.91±1.37 | 0.01±1.39 | 0.9 |
| Stroke volume (ml) | 60±16 | 64±23 | 4±16 | 0.4 |
| Heart rate (beats/min) | 85±16 | 86±13 | 1±10 | 0.7 |

Data are means±SD.

**Supplementary Table 4. Effect of submaximal exercise on measurements of cardiac output, stroke volume and heart rate.**

| **Parameter** | **Rest** | **20rpm** | **35rpm** | **Overall P** |
| --- | --- | --- | --- | --- |
| Cardiac output (L/min) | 5.1±0.8 | 7.4±0.3 | 9.0±1.9 | P<0.001 |
| Stroke volume (ml) | 72±19 | 93±19 | 101±26 | P<0.001 |
| Heart rate (beats/min) | 74±15 | 81±11 | 89±22 | P=0.03 |

Data are means±SD.
